# Analysis of Sodium Chloride Intake and Treg/Th17 Lymphocytes in Healthy Individuals and Patients with Rheumatoid Arthritis or Systemic Lupus Erythematosus

**DOI:** 10.1155/2018/9627806

**Published:** 2018-07-09

**Authors:** Marlen Vitales-Noyola, Esther Layseca-Espinosa, Lourdes Baranda, Carlos Abud-Mendoza, Perla Niño-Moreno, Adriana Monsiváis-Urenda, Yvonne Rosenstein, Roberto González-Amaro

**Affiliations:** ^1^Research Center for Health Sciences and Biomedicine, UASLP, 78210 San Luis Potosí, SLP, Mexico; ^2^Department of Immunology, School of Medicine, UASLP, 78210 San Luis Potosí, SLP, Mexico; ^3^Regional Unit of Rheumatology and Osteoporosis, Hospital Central Dr. Ignacio Morones Prieto, 78210 San Luis Potosí, SLP, Mexico; ^4^Faculty of Chemical Sciences, UASLP, 78210 San Luis Potosí, SLP, Mexico; ^5^Instituto de Biotecnología, UNAM, 62210 Cuernavaca, MOR, Mexico

## Abstract

We assessed different immune parameters in patients with rheumatoid arthritis (RA) and systemic lupus erythematosus (SLE) with low (LSI) and high (HSI) sodium intake. Thirty-eight patients with RA, thirty-seven with SLE, and twenty-eight healthy subjects were studied and classified as LSI or HSI. Levels and suppressive function of CD4^+^CD25^+^Foxp3^+^ and CD4^+^CD69^+^Foxp3^−^ Treg cells were determined by flow cytometry in blood samples. Levels and *in vitro* differentiation of Th17 cells were also assessed. Similar levels of CD4^+^CD25^+^Foxp3^+^ and CD4^+^CD69^+^Foxp3^−^ Treg cells were observed in LSI and HSI patients or controls. However, a positive correlation was detected between sodium intake and levels of CD4^+^CD25^+^Foxp3^+^ Treg cells in SLE and a negative association between CD4^+^CD69^+^Foxp3^−^ Treg cells and sodium intake in RA. No other significant associations were detected, including disease activity and sodium intake. Moreover, the suppressor activity of CD4^+^CD25^+^Foxp3^+^ and CD4^+^CD69^+^Foxp3^−^ Treg cells was similar in LSI and HSI patients or controls. The levels and *in vitro* differentiation of Th17 cells were also similar in LSI and HSI individuals. Our results suggest that, in the population studied (Mexican mestizo), the level of sodium intake is not apparently associated with different relevant immune parameters in healthy subjects or patients with SLE or RA.

## 1. Introduction

Human autoimmune diseases are complex disorders that arise from the interactions between polygenic risk factors, environmental influences, and defects in immune regulatory mechanisms. Rheumatoid arthritis (RA) is an autoimmune chronic disease characterized, among others, by pain, inflammation, and destruction of diarthrodial joints, resulting in functional disability [1]. Systemic lupus erythematosus (SLE) is an autoimmune condition characterized by the synthesis of many different autoantibodies, deposition of immune complexes, and inflammation of several tissues and organs [2]. In both conditions, aberrations in the immune modulatory mechanisms, including the number and function of T regulatory (Treg) cells have been described [3–5].

Treg cells are able to inhibit the activation and proliferation of effector lymphocytes, and their activity has an important role in the pathogenesis of autoimmune and chronic inflammatory diseases [6]. Several Treg cell subsets have been characterized, including the CD4^+^CD25^high^ natural nTreg lymphocytes expressing the forkhead box P3 transcription factor (Foxp3), which exert their inhibitory effect through different mechanisms, including the synthesis of regulatory cytokines, including the transforming growth factor-*β* (TGF-*β*) and interleukins-10 and -35 (IL-10 and IL-35, resp.) [6]. CD4^+^Foxp3^+^ Treg cells may differentiate in the thymus (tTreg cells), in the peripheral lymphoid tissues (pTreg cells), or *in vitro* (induced or iTreg cells) [6]. Different data indicate the relevant role of these CD4^+^Foxp3^+^ Treg cells in the pathogenesis of autoimmune diseases, including the congenital deficiency of Foxp3, which results in the IPEX syndrome (immune dysregulation, autoimmune polyendocrinopathy, and inflammatory enteropathy) [7]. In addition, alterations in the number and function of CD4^+^Foxp3^+^ Treg cells have been reported in most autoimmune diseases as well as in other immune-mediated conditions [4–6, 8, 9]. Accordingly, abnormal number and function of CD4^+^Foxp3^+^ Treg cells have been reported in RA and SLE, and it has been proposed that the autoimmune responses observed in these patients could be controlled by the clinical use of Treg cells to treat these conditions [4–6].

Other regulatory lymphocyte subsets have been described, including Tr1, Tr35, and Th3 lymphocytes [10, 11]. In addition, we and others have detected a subset of T cells that show a constitutive expression of CD69 (with a CD4^+^CD69^+^Foxp3^−^TGF-*β*^+^IL-10^+^ phenotype and a variable expression of CD25) and that exerts a relevant *in vitro* suppressive effect on the activation of autologous effector T cells [12, 13]. These CD69^+^ Treg cells also show an abnormal number and function in patients with different conditions, including autoimmune thyroid diseases, SLE, and liver carcinoma [14–16]. Moreover, it has been described that CD4^+^NKG2D^+^ T cells with regulatory activity and primed to produce IL-10 are detected in normal individuals and that patients with juvenile-onset SLE show increased levels of these lymphocytes [17]. In addition, we have observed that a fraction of this cell subset corresponds to CD4^+^CD69^+^ Treg lymphocytes [13] and that patients with autoimmune thyroid disease or SLE show abnormal numbers and function of these cells [14, 15].

T helper 17 (Th17) lymphocytes are mainly characterized by the synthesis of IL-17A, IL-17F, and IL-22, and their differentiation is induced by a set of cytokines, including IL-1*β*, IL-6, and IL-23. It has been described that Th17 cells are clearly involved in the pathogenesis of and the inflammatory phenomenon observed in different conditions, including RA, Crohn's disease, and multiple sclerosis (MS) [18, 19]. Accordingly, the cytokines that induce this cell subset as well as the cytokines synthesized by them have become therapeutic targets for the treatment of several inflammatory immune-mediated conditions [20].

It has been shown that high-salt (NaCl) concentrations favor the differentiation of Th17 lymphocytes, both *in vivo* and *in vitro* [21]. Accordingly, a high-salt intake (HSI) is significantly associated with the severity of experimental autoimmune encephalomyelitis (EAE), an animal model of MS [22]. Likewise, it has been reported that those MS patients with HSI show a most severe form of the disease compared to patients with low-salt intake (LSI) [22]. Similar findings have been observed in MRL/lpr mice, an animal model of SLE [23]. In addition, Scrivo et al. observed that a low-sodium dietary regimen is associated with a significant reduction of CD4^+^CD45RA^−^Foxp3^low^ lymphocytes in patients with RA as well as with a reduction in Th17 cells with an enhancement in Foxp3^+^ Treg cells in patients with SLE [24]. The mechanisms involved in the induction of Th17 cells by high-NaCl concentrations have been characterized. Thus, it has been reported that high concentrations of sodium induce the activation of the p38 protein kinase and the nuclear factor NFAT5 as well as the synthesis and activation of the serum and glucocorticoid-regulated kinase 1 or SGK1; this kinase in turn induces the expression of the receptor for IL-23 and thus favors the differentiation of Th17 cells and the release of proinflammatory cytokines [25]. Moreover, it has been reported that high-sodium concentrations interfere with the differentiation and function of CD4^+^Foxp3^+^ Treg cells [26] that favors the differentiation and activation of proinflammatory M1-type macrophages [27]. Overall, these data have strongly suggested that high-salt intake is causally associated with the pathogenesis of different chronic inflammatory conditions, through different mechanisms.

In order to further assess the role of NaCl intake in the pathogenesis of inflammatory immune-mediated diseases, we decided to analyze different clinical and laboratory parameters in healthy controls and patients with SLE and RA, classified as LSI and HSI. Our results suggest that the level of sodium intake does not seem to be significantly associated with different relevant immune parameters in either healthy subjects or patients with the immune-mediated diseases analyzed.

## 2. Materials and Methods

### 2.1. Aim and Design

The aim of this study was to explore the possible association between the level of salt intake and different immune parameters in healthy individuals and patients with SLE and RA. A cross-sectional observational and experimental study was carried out. This study was done at the Research Center for Health Sciences and Biomedicine, UASLP, and the Regional Unit of Rheumatology and Osteoporosis, Hospital Central Dr. Ignacio Morones Prieto, San Luis Potosí, SLP, México.

### 2.2. Individuals

Thirty-eight patients with RA (36 females and 2 males), according to the criteria of the American College of Rheumatology, and a mean age of 42.6 ± 10.5 years were included in the study (Table 1). According to the DAS28 index, at the time of the study, twenty patients had low activity (DAS28 ≤ 4) and eighteen had high activity (DAS28 > 4). Twenty-eight patients were receiving prednisone (2.5 to 5.0 mg/day) and/or disease-modifying antirheumatic drugs (methotrexate 7.5 to 20.0 mg/week and/or sulfasalazine 1.0 to 3.0 g/day) at the time of study; thus, seven patients were under methotrexate monotherapy; four were receiving a combination of methotrexate, prednisone, and sulfasalazine; sixteen methotrexate and prednisone; and one patient was under sulfasalazine monotherapy. No patients under therapy with biological agents were included in the study. Ten patients were untreated at the time of study. Thirty-seven patients with SLE, according to the classification criteria of the American College of Rheumatology, were also studied (Table 2). Thirty-four patients were females and three males, with a mean age of 36.5 ± 15.5 years. According to the MEX-SLEDAI score, 75% of patients had a moderate-to-severe active disease and 25% were in remission. Twenty-eight patients were receiving prednisone (2.5 to 7.5 mg/day) and/or immunosuppressive drugs (methotrexate 10.0 to 15.0 mg/week and/or azathioprine 50–100 mg/day); thus, four patients were under methotrexate monotherapy; fifteen were receiving methotrexate and prednisone; seven methotrexate, prednisone, and azathioprine; and two prednisone and azathioprine. Nine patients were untreated at the time of study, and no patients with evidence of renal failure or receiving biological agents were studied. Twenty-eight healthy subjects (twenty-four females and four males) with a mean age of 36.3 ± 12.1 years were also studied. This study was approved by the Bioethical Committee of the Hospital Central Dr. Ignacio Morones Prieto, and all individuals included in it signed an informed consent.

### 2.3. Estimation of Sodium Chloride Intake

Salt intake was estimated through two different parameters, the measurement of sodium excretion by urine and a written questionnaire. In the first case, NaCl intake was calculated through 24-hour urinary sodium excretion, by using the following formula: NaCl = Na (g/day) × 100/39.3 [28]. In addition, a written questionnaire of alimentary habits, validated by the World Health Organization (WHO) and the Pan-American Health Organization (PAHO), was employed [29, 30]. The latter one was adapted (by including three additional foods and by considering the salt added to the foods with the salt shaker during the meal) for the population of our study (Mexican mestizos), and the results were compared with those obtained using the urinary sodium excretion. According to these instruments, the individuals that had been included in the study were subsequently classified as having LSI (less than 5.0 g/day of NaCl) or HSI (5.0 or more g/day of NaCl). Those individuals with a discordance greater than 3.0 g/day of salt intake between the values obtained with the questionnaire and sodium excretion were excluded from the study.

### 2.4. Flow Cytometry Analysis

Peripheral blood mononuclear cells (PBMC) were isolated by Ficoll-Hypaque (GE Healthcare Life Sciences, Issaquah, WA) density-gradient centrifugation, and cellular viability was evaluated by trypan blue stain. When indicated, cells were incubated in RPMI-1640 culture medium (Gibco by Life Technologies, Gaithersburg, MD) supplemented with 10% fetal bovine serum, glutamine (2.0 mM), and penicillin (100 u/ml)/streptomycin (100 *μ*g/ml) for 4 hours at 37°C with 5% CO_2_. Brefeldin (10 *μ*g/ml) was also added. Then, cells were washed and stained with the following monoclonal antibodies (mAbs): CD4/FITC or CD4/APC-Cy7 (BioLegend, San Diego, CA), CD25/APC-Cy7 (BD Biosciences, San Jose, CA), NKG2D/FITC (eBioscience), anti-LAP/PerCp-Cy5.5 (BioLegend), and CD69/APC (eBioscience). Then, cells were fixed and permeabilized with the Foxp3 Fix/Perm kit (eBioscience) and additionally stained with mAbs against IL-10 (PE) (BioLegend) and Foxp3 (PE-Cy7) (eBioscience). Thus, as previously reported [13], when CD69^+^ Treg cells were analyzed, the following strategy was employed: 1st dot plot: CD4/FITC and CD25/APC-Cy7; 2nd dot plot: LAP/PerCp-Cy5 and CD69/APC; and 3rd dot plot: Foxp3/PE-Cy7 and IL-10/PE. In the case of CD69^+^NKG2D^+^ Treg cells, the analysis strategy was as follows: 1st dot plot: CD4/APC-Cy7 and NKG2D/FITC; 2nd dot plot: LAP/PerCp-Cy5 and CD69/APC; and 3rd dot plot: Foxp3/PE-Cy7 and IL-10/PE. Moreover, other cells were stained with CD4/FITC (eBioscience) and fixed with p-formaldehyde 4% and permeabilized with saponin 0.1% and stained with mAbs against IL-17/PE (eBioscience) and IL-22/APC (eBioscience). Data were acquired in FACSCanto II flow cytometer (Becton Dickinson) and analyzed using FlowJo version 10 (Tree Star). According to these analyses, Foxp3^+^ Treg cells corresponded to CD4^+^CD25^high^Foxp3^+^ lymphocytes, CD69^+^ Treg cells to CD4^+^CD25^var^CD69^+^LAP^+^IL-10^+^Foxp3^−^ lymphocytes, CD69^+^NKG2D^+^ Treg cells to CD4^+^NKG2D^+^CD69^+^LAP^+^IL-10^+^Foxp3^−^, and Th17 cells to CD4^+^IL-17A^+^IL-22^+^ lymphocytes. In some cases, CD4^+^NKG2D^+^ double-positive cells were also analyzed, which correspond to a fraction of CD69^+^NKG2D^+^ Treg cells and to a subset of CD4^+^ lymphocytes with no regulatory activity [17].

### 2.5. Functional Analysis of CD25^+^ and CD69^+^ Treg Cells

The suppressive function of CD69^+^ Treg lymphocytes was assessed by an assay of inhibition of cell activation [31]. In these assays, one aliquot of PBMC was depleted from CD69^+^ lymphocytes by magnetic negative selection using a MACS cell separation system (Miltenyi Biotec Inc., St Louis, MO). Briefly, PBMC were incubated with an anti-CD69 mAb (eBioscience) for 30 minutes, washed and then incubated with paramagnetic rat anti-mouse IgG MicroBeads (Miltenyi Biotec), and washed again. Afterwards, cells were poured onto MS columns (Miltenyi Biotec), and CD69-negative cells were recovered. Then, these CD69-depleted cells as well as an aliquot of whole PBMC were stimulated by culturing in 24-well plates (Costar) precoated for 1.5 h with an anti-CD3 (OKT3 clone, 5.0 *μ*g/ml) and an anti-CD28 (clone 28.2, 5.0 *μ*g/ml) mAb at 37°C, 5% CO_2_. Finally, cells were incubated in RPMI-1640 medium supplemented with 10% FBS, glutamine, and penicillin/streptomycin for 7 hours at 37°C with 5% CO_2_. At starting the cell culture, an anti-CD40L/PE mAb (BD Biosciences) was added, and at the end of incubation, cells were washed and analyzed for CD40L expression in a FACSCanto II flow cytometer (Becton Dickinson) and data processed with the FlowJo software (Tree Star). In the case of assays for CD4^+^CD25^high^Foxp3^+^ cell activity, we employed the same procedure but using an anti-CD25 mAb (eBioscience) instead of an anti-CD69. In both cases, the suppressor activity was calculated by comparing the percent of CD40L^+^ cells in the two aliquots of cells, as follows: %inhibition = 100 − [(%CD40L^+^ cells in whole PBMC)/(%CD40L^+^ cell cultures depleted from CD69^+^ cells) 100].

### 2.6. In Vitro Differentiation of Th17 Cells

PBMC from patients or healthy controls were activated with plate-bound anti-CD3/CD28 (5.0 *μ*g/ml) and grown in IMDM culture medium supplemented with 10% fetal bovine serum, glutamine (2.0 mM), and penicillin (100 u/ml)/streptomycin (100 *μ*g/ml) and 5% CO_2_ at 37°C. These cells were induced to differentiate or not towards the Th17 lineage by adding 8 ng/ml TGF-*β*, 10 ng/ml IL-1*β*, 50 ng/ml IL-6, 10 ng/ml IL-23, and 10 *μ*g/ml anti-IL-4 mAb for 6 days. Three hours before its harvesting, cells were restimulated with a leukocyte activation cocktail with GolgiPlug (BD Pharmingen). Finally, cells were collected, washed, and analyzed for the presence of CD4^+^IL-17A^+^ lymphocytes, as stated above.

### 2.7. Quantification of Cytokines

Cell cultures similar to those employed to test the suppressive function of Treg cells on T cell activation were run but in the absence of an anti-CD40L mAb and incubated for 24 h. At the end of the incubation, cell-free supernatants were obtained and the levels of the indicated cytokines were determined by flow cytometry using a Cytometric Bead Array (BD Biosciences). The following cytokines were analyzed: IL-2, IL-4, IL-6, IL-10, TNF-*α*, IFN-*γ*, and IL-17A. Data analysis was performed by using the FCAP Array Software v3.0 (BD Biosciences).

### 2.8. Statistical Analysis

Data with normal distribution were presented as the arithmetic mean and SD, and data with a non-Gaussian distribution were presented as the median and interquartile range (Q_1_–Q_3_). Analysis of 2 groups was performed with the Mann–Whitney *U* test and comparisons among 3 groups with the Kruskal-Wallis sum-rank test. Data were analyzed using the Graph Pad Prism 5 software, and *p* values < 0.05 were considered as significant.

## 3. Results

**Figure 1:**
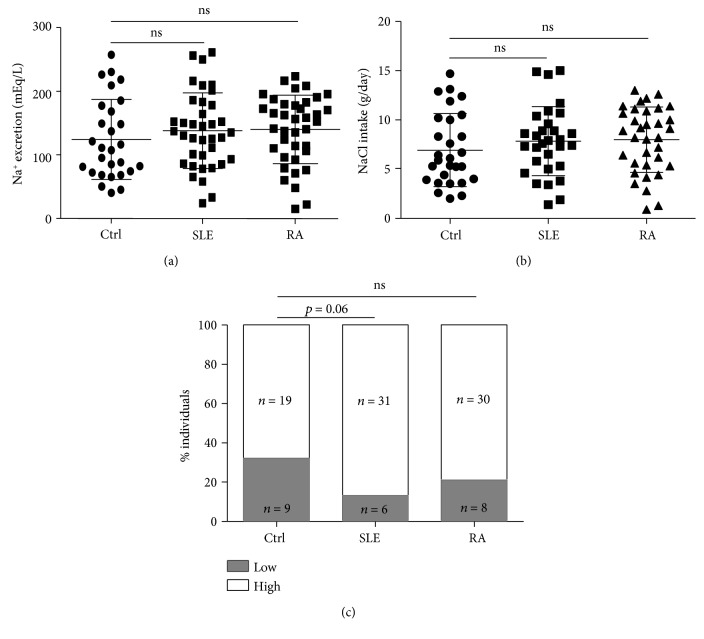
Estimation of salt intake in controls and patients. (a, b) Sodium excretion and salt intake was estimated in healthy controls (Ctrl) and patients with SLE or RA, as stated in the Materials and Methods. No significant differences were observed among the three groups. Horizontal lines correspond to the arithmetic mean and SD. (c) Proportions of individuals with low (less than 5.0 g/day of NaCl) and high (5.0 g/day of NaCl or more) salt intake in healthy controls (Ctrl) and patients with SLE and RA. ns: nonsignificant.

As stated in the Materials and Methods, the salt intake was estimated in the individuals included in this study and they were classified as LSI (less than 5.0 g/day of NaCl) and HSI (5.0 g/day of NaCl or more). As shown in Figure 1, sodium excretion (mEq/L) and sodium intake (g/day) were similar in the three groups studied. In addition, when the proportions of LSI and HSI individuals in each group were compared, no significant differences were observed. However, the percent of individuals with LSI tended to be higher in the healthy control group (*p* = 0.060 compared to SLE, Fisher exact test, Figure 1(c)).

**Figure 2:**
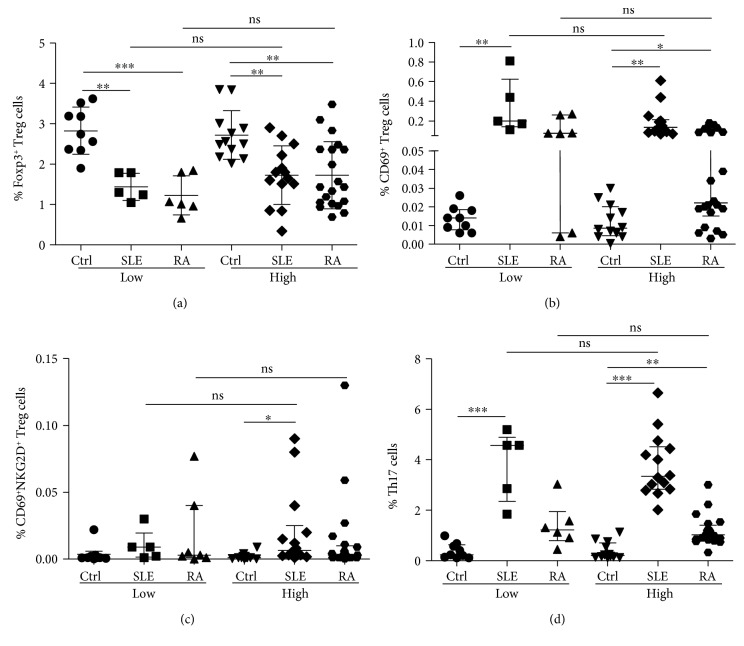
Levels of Treg and Th17 cells in the peripheral blood from healthy subjects and patients with SLE or RA with low- and high-salt intake. The frequency of Foxp3^+^ and CD69^+^ Treg cells and Th17 lymphocytes was determined by multiparametric flow cytometry analysis in blood samples from healthy controls and patients with SLE or RA, with low- or high-salt intake. (a) Percent of CD4^+^CD25^high^Foxp3^+^ cells. (b) Frequency of CD4^+^CD25^var^CD69^+^LAP^+^IL-10^+^Foxp3^−^ Treg cells. (c) Percent of CD4^+^NKG2D^+^CD69^+^LAP^+^IL-10^+^Foxp3^−^ Treg cells. (d) Frequency of CD4^+^IL-17^+^ lymphocytes. All percentages are referred to total lymphocytes. Data correspond to the arithmetic mean and SD (a) or the median and IQR (b–d). ^∗^*p* < 0.05; ^∗∗^*p* < 0.01; ^∗∗∗^*p* < 0.001; ns: nonsignificant.

As it has been previously reported, when we analyzed the levels of CD4^+^CD25^high^Foxp3+ Treg cells, significant lower levels of these lymphocytes were observed in patients with SLE or RA compared to healthy controls, both in the case of LSI or HSI individuals (*p* < 0.05 in all cases, Figure 2(a)). However, we did not detect significant differences when LSI and HSI individuals were compared, in either healthy subjects or patients with SLE or RA (*p* > 0.05 in all cases, Figure 2(a)). In the case of CD69^+^ Treg cells, significant enhanced levels of these lymphocytes (CD4^+^CD25^var^CD69^+^LAP^+^IL-10^+^Foxp3^−^) were observed in SLE and RA patients compared to controls, both in the case of LSI or HSI individuals (*p* < 0.05 in all cases, Figure 2(b)). Nevertheless, as in the case of Foxp3^+^ Treg cells, similar levels of CD69^+^ Treg lymphocytes were observed when LSI and HSI were compared, in the three groups studied (*p* > 0.05 in all cases, Figure 2(b)). Likewise, no significant differences were detected between LSI and HSI individuals in the case of the CD69^+^NKG2D^+^ Treg cell subset levels, in all groups studied (*p* > 0.05 in all cases, Figure 2(c)). Moreover, although increased levels of peripheral blood Th17 cells were observed in patients with SLE or RA compared to healthy controls (*p* < 0.05 in all cases, Figure 2(d)), similar levels of this helper cell subset were observed when LSI and HSI individuals were compared, in the three groups studied (*p* > 0.05 in all cases, Figure 2(d)).

**Figure 3:**
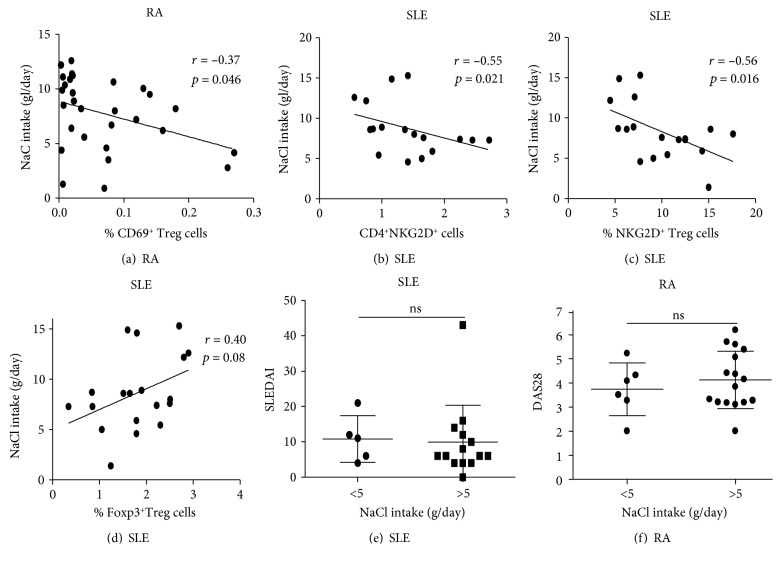
Correlation analysis between salt intake and clinical and laboratory parameters in patients with SLE and RA. Patients with RA showed a significant negative correlation between salt intake and % of CD69^+^ Treg cells (CD4^+^CD25^var^CD69^+^LAP^+^IL-10^+^Foxp3^−^) (a), whereas in patients with SLE, a significant negative association of salt intake and the levels of CD4^+^NKG2D^+^ (b) or NKG2D^+^ Treg cells (CD4^+^NKG2D^+^CD69^+^LAP^+^IL-10^+^Foxp3^−^) (c) was observed. Although in SLE patients salt intake tended to be associated with the % of Foxp3^+^ Treg cells, the value of *r* was nonsignificant (d). Both patients with SLE (e) and RA (f) showed similar levels of disease activity in those cases with low- and high-salt intake. ns: nonsignificant. (e, f) Data correspond to the arithmetic mean and SD.

When an association analysis was performed, a significant negative correlation between NaCl intake and the percent of CD69^+^ Treg cells was observed in patients with RA (*r* = −0.37, *p* = 0.046, Figure 3(a)). Furthermore, a significant negative association between NaCl intake and the levels of CD4^+^NKG2D^+^ cells was observed in patients with SLE (*r* = −0.55, *p* = 0.02, Figure 3(b)). A similar result was observed in the case of CD69^+^NKG2D^+^ Treg lymphocytes (*r* = −0.56, *p* = 0.016, Figure 3(c)). Conversely, the levels of Foxp3^+^ Treg cells tended to be positively associated with NaCl intake in patients with SLE; however, a no significant value of *p* was obtained in this case (*r* = 0.04, *p* = 0.08, Figure 3(d)). Likewise, no apparent association was detected between the levels of Th17 cells and NaCl intake in the three groups studied (*p* > 0.05 in all cases, data not shown). Furthermore, LSI and HSI patients with SLE or RA showed similar levels of disease activity, according to the SLEDAI and DAS28 indices, respectively (*p* > 0.05 in both cases, Figures 3(e) and 3(f)). Moreover, an additional analysis showed no apparent association between the therapies that the patients were receiving and the different parameters measured in the study (data not shown). Accordingly, when these immune parameters were compared in treated and untreated patients (with SLE or RA), no significant differences were observed (data not shown).

**Figure 4:**
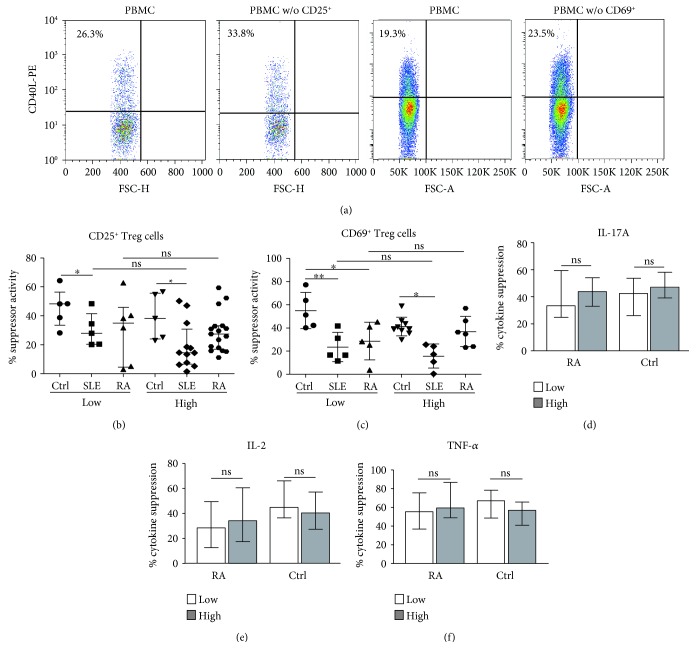
Functional analysis of Treg lymphocytes in healthy controls and patients with SLE or RA, with low- and high-salt intake. PBMC were depleted or not of CD69^+^ or CD25^+^ cells, stimulated through CD3/CD28 for 7 h, and then, the expression of CD40L was assessed by flow cytometry, as indicated in the Materials and Methods. (a) Flow cytometry dot plots showing the expression of CD40L in unfractionated and Treg cell-depleted PBMC stimulated through CD3/CD28. These data correspond to cells from a HSI healthy control. (b) Suppressor activity (calculated as stated in the Materials and Methods) of CD25^+^ Treg cells in healthy controls (Ctrl) and patients with SLE or RA, with low- or high-sodium intake. (c) Suppressor activity of CD69^+^ Treg cells in healthy controls (Ctrl) and patients with SLE or RA, with low- or high-sodium intake. (d–f) In separate experiments, PBMC were depleted or not of CD69^+^ or CD25^+^ cells and stimulated through CD3/CD28 for 24 h, and then, the levels of the indicated cytokines were analyzed in cell supernatants as stated in the Materials and Methods. Data from patients with RA and healthy controls, with low- and high-sodium intake, are shown. ^∗^*p* < 0.05; ^∗∗^ *p* < 0.01; ns: nonsignificant. Data correspond to the median and IQR.

We then analyzed the function of two subsets of Treg lymphocytes. As shown in Figure 4(a), the function of CD4^+^CD25^+^Foxp3^+^ and CD4^+^CD69^+^ Treg cells was assessed through assays of inhibition of lymphocyte activation (expression of CD40L) and suppression of cytokine release. In the former assay, we observed that patients with SLE showed a significant diminution in the suppressor activity of CD4^+^CD25^+^Foxp3^+^ cells compared to healthy controls (*p* < 0.05, Figure 4(b)). A similar trend was observed in the case of RA, but in this case, a significant difference was not reached (Figure 4(b)). Moreover, when the activity of these Treg cells was compared in LSI and HSI individuals, we did not detect significant differences in any group studied (*p* > 0.05 in all cases, Figure 4(b)). As shown in Figure 4(c), similar results were observed for the activity of CD4^+^CD69^+^ Treg cells, with no significant differences between LSI and HSI individuals, in either healthy controls or patients with RA or SLE (*p* > 0.05). Accordingly, when we analyzed the suppression of cytokine release by Treg cells, we observed similar levels of inhibition in LSI and HSI individuals, in the three groups studied and for all cytokines analyzed (IL-2, IL-4, IL-6, IL-10, TNF-*α*, IFN-*γ*, and IL-17A) (Figures 4(d)–4(f) and data not shown).

**Figure 5:**
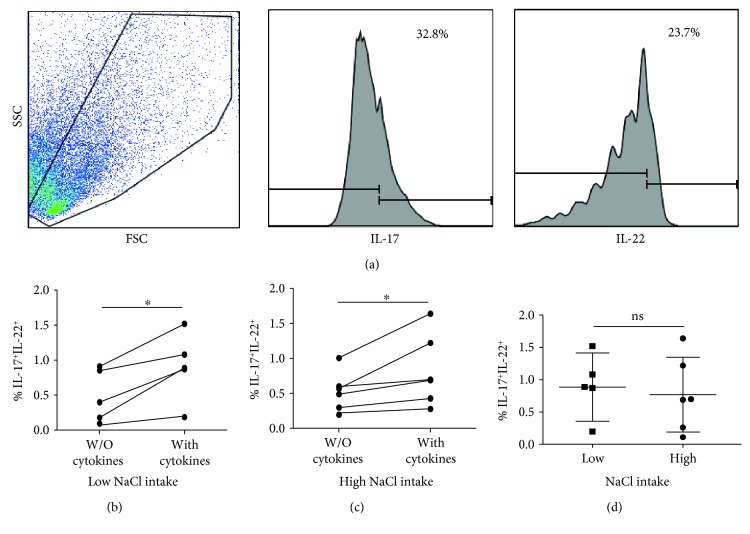
*In vitro* differentiation of Th17 cells in healthy controls and patients with RA, with low- and high-salt intake. PBMC were cultured for seven days in the presence or not of a cytokine cocktail to induce the differentiation of Th17 cells, as described in the Materials and Methods. (a) Flow cytometry analysis of CD4^+^IL-17A^+^IL-22^+^ cells. A representative dot plot (forward versus side scatter) and histograms of IL-17 and IL-22 expression are shown. Data correspond to a patient with RA with high-salt intake. (b, c**)** Percent of Th17 cells in PBMC cultures with the addition or not of a cytokine cocktail that induces the differentiation of these cells. Data correspond to patients with RA with low- and high-salt intake, as indicated. (d) Comparison of the percent of Th17 cells induced *in vitro* in PBMC from RA patients with low- and high-salt intake. Data correspond to the arithmetic mean and SD. ^∗^*p* < 0.05.

We also analyzed the *in vitro* differentiation of CD4 T naïve cells into Th17 lymphocytes in healthy individuals and patients with RA or SLE with low- and high-sodium intake. Although in these assays we only observed a modest (but significant) differentiation of Th17 cells, we observed that similar levels of these cells were generated in LSI and HSI individuals, in the three groups studied (*p* > 0.05 in all cases, Figure 5 and data not shown).

Finally, since we only performed a single determination of NaCl excretion to all individuals included in the study, we decided to further analyze our results after the exclusion of those patients and controls with sodium intake levels near to the cutoff value (5.0 g/day) employed by us. Under such conditions, five patients with RA, four with SLE, and five controls, with daily sodium intake between 4.0 and 6.0 g, were excluded. These analyses showed similar results, with no significant differences between these LSI (<4.0 g/day) and HSI (>6.0 g/day) groups, regarding the levels of Foxp3+ and CD69+ Treg cells, their suppressive activity, and the number and *in vitro* differentiation of Th17 lymphocytes (data not shown). In addition, we also analyzed the results of this study after classifying healthy controls and patients by tertiles of sodium intake. In this case, similar results were obtained, with no significant differences among the three categories of salt intake (low, high, and very high) in the three groups studied (data not shown).

## 4. Discussion

Different factors determine the loss of immune tolerance to self-antigens and the appearance of chronic inflammatory autoimmune disease, mainly genetic and environmental factors as well as abnormalities in the immune regulatory mechanisms and, very likely, the gut microbiota [1–6]. In recent years, different very interesting reports have indicated the important influence of sodium concentration and salt intake on the innate and adaptive immune responses. Thus, it has been described that high-sodium concentrations (that can be detected in the interstitial space of different tissues and organs, including the lymph nodes, skin, and renal medulla) are able to induce the differentiation of Th17 cells [21], which are involved in the pathogenesis of several inflammatory immune-mediated diseases, including RA and SLE [32]. In addition, it has been shown that high-NaCl concentrations favor the differentiation of proinflammatory type M1 macrophages, which also have a key role in the pathogenesis of the tissue damage associated to autoimmunity [27]. Furthermore, it has been observed that sodium chloride inhibits the suppressive activity of Foxp3^+^ Treg lymphocytes [26]. Accordingly, it has been proposed that high-salt intake may have a relevant role in the induction and maintenance of autoimmune conditions, including RA, SLE, and MS [25, 33, 34].

In apparent contrast with all the above information, we have observed that healthy individuals as well as patients with SLE or RA, with low- and high-salt intake, show similar levels of Foxp3^+^ and CD69^+^ Treg cells in the peripheral blood. In addition, no significant differences were detected in the suppressive activity of these Treg cell subsets in those individuals with low and high dietary sodium. Similar results were obtained regarding the proportion of Th17 cells in the peripheral blood of the three groups studied as well as in the assays of the *in vitro* differentiation of these lymphocytes. Finally, no apparent association was observed between disease activity and the level of sodium intake either in SLE or in RA patients.

Although we do not have a conclusive explanation for the apparent contradiction between our results and the previous reports regarding the effects of high-sodium concentration on Treg cell function and Th17 lymphocyte differentiation, it is worth mentioning that the laboratory parameters studied by us had not been analyzed in previous studies, comparing LSI and HSI subgroups of individuals. In this regard, the interesting work of Hernandez et al., which demonstrates the inhibitory effect of high-sodium concentrations on the activity of human Foxp3+ Treg cells [26], was performed in a xenogenic graft-versus-host disease model. Moreover, in the study of Yi et al., showing that low-salt diet is associated with a diminished production of proinflammatory cytokines [34], no *in vitro* experiments of cytokine release were performed. Likewise, in the report of Wen et al., the induction of IL-17 by HSI in healthy subjects (and its reversion by potassium supplementation) was assessed by measuring the mRNA levels of this cytokine in PBMC and the protein in plasma, with no *in vitro* assays of cytokine synthesis [35]. Therefore, it is possible that the apparent contradiction between our results and previous reports on the effect of HSI on Treg and Th17 cells in humans might be due to methodological differences. In this regard, although our results may not be considered conclusive, we think that this study further supports that the possible role of HSI on the pathogenesis of both SLE and RA could be rather complex. In this regard, it is of interest the study of Kuek et al., which detected an apparent lack of association between the level of sodium intake and the development of RA in nonsmoker individuals [32]. Accordingly, it has been reported that high-sodium consumption is significantly associated with the presence of anticitrullinated peptide antibodies only among smoker patients with RA [36]. Likewise, although in the MRL/lpr mice model of SLE HSI increases the severity of nephritis [23], this effect is not observed in the female NZBWF1 mice model of SLE [37]. In addition, in the recent study of Scrivo et al., a nonsignificant effect of a low-sodium dietary regimen on Th17 and Foxp3^+^ Treg cell levels was observed in patients with RA (however, significant changes in the proportions of these cell subsets were detected in patients with SLE) [24]. Accordingly, different authors have commented that it could be premature to state, at this time, that dietary salt influences autoimmune disease in humans [25, 38, 39]. In this regard, it is worth mentioning that there are also controversial results in the case of the possible association between sodium intake (or urinary excretion) and cardiovascular events in normotensive individuals [40, 41]. However, in the case of multiple sclerosis, there are additional data that support the association between HSI and disease severity [27, 42].

Different factors may influence the results obtained in this pilot study. The first one is the relatively small proportion of individuals, in the three groups studied, with LSI. This was due to the study design, which stated that patients and controls should be consecutively recruited, and then the salt intake should be determined. Other factors may correspond to the genetic background of the population studied (Mexican mestizo), the diet, the degree of sun exposure and vitamin D levels, and so on. In any case, we consider that the data of this cross-sectional pilot study strongly suggest that, in order to further define the precise role of salt intake in the pathogenesis of SLE and RA, it would be very convenient to perform a cohort study with a great number of patients with a serial analysis of different clinical and laboratory parameters.

In conclusion, data of this cross-sectional pilot study suggest that the degree of salt intake does not seem to be significantly associated with the levels and function of at least two Treg cell subsets and the proportion of Th17 lymphocytes in the peripheral blood from healthy subjects or patients with SLE or RA. Since these data do not support the putative role of sodium intake on the pathogenesis of immune-mediated inflammatory diseases, we consider that it would be very convenient to confirm these results through a longitudinal study with a great number of individuals.

## Figures and Tables

**Table 1:** Main clinical and laboratory characteristics of patients with rheumatoid arthritis.

Number (female/male)	38 (36/2)
Mean age (range)	42.5 ± 10.5 years (19–61)
Disease duration (range)	5.4 ± 4.7 years (0–10.5)
DAS28 > 4 (%)	18/38 (47%)
Therapy^∗^	
Prednisone (dose range)	20/38 (2.5–5.0 mg/day)
Methotrexate (dose range)	27/38 (7.5–20.0 mg/week)
Sulfasalazine (dose range)	5/38 (1.0–3.0 g/day)
Biological agents	0/38
Untreated patients (%)	10/38 (26%)
Anticitrullinated peptide antibodies	37/38
Salt intake	
Low (range)	8/38 (1.7–4.9 g/day)
High (range)	30/38 (5.0–13.5 g/day)

^∗^Most treated patients were receiving two or more drugs.

**Table 2:** Main clinical and laboratory characteristics of patients with systemic lupus erythematosus.

Number (female/male)	37 (34/3)
Mean age (range)	36.5 ± 15.5 years (18.3–52.5)
Disease duration (range)	8.3 ± 6.7 years (0.4–16.3)
MEX-SLEDAI > 4.0	28/37 (75.6%)
Therapy^∗^	
Prednisone(dose range)	24/37 (2.5–7.5 mg/day)
Methotrexate (dose range)	26/37 (10.0–15.0 mg/week)
Azathioprine (dose range)	9/37 (50.0–100.0 mg/day)
Biological agents	0/37
Untreated patients (%)	9/31 (29%)
Antinuclear antibodies (%)	37/37 (100%)
Low complement levels	25/37 (68%)
Salt intake	
Low (range)	6/37 (1.8–4.6 g/day)
High (range)	31/37 (5.0–15.1)

^∗^Most treated patients were receiving two or more drugs.

## Data Availability

The data used to support the findings of this study are available from the corresponding author upon request.

## References

[1] Angelotti F., Parma A., Cafaro G., Capecchi R., Alunno A., Puxeddu I. (2017). One year in review 2017: pathogenesis of rheumatoid arthritis. *Clinical and Experimental Rheumatology*.

[2] Podolska M. J., Biermann M. H., Maueröder C., Hahn J., Hermann M. (2015). Inflammatory etiopathogenesis of systemic lupus erythematosus: an update. *Journal of Inflammation Research*.

[3] Moradi B., Schnatzer P., Hagmann S. (2014). CD4^+^CD25^+^/highCD127low/^−^ regulatory T cells are enriched in rheumatoid arthritis and osteoarthritis joints—analysis of frequency and phenotype in synovial membrane, synovial fluid and peripheral blood. *Arthritis Research & Therapy*.

[4] Alvarado-Sánchez B., Hernández-Castro B., Portales-Pérez D. (2006). Regulatory T cells in patients with systemic lupus erythematosus. *Journal of Autoimmunity*.

[5] Vigna-Pérez M., Abud-Mendoza C., Portillo-Salazar H. (2005). Immune effects of therapy with adalimumab in patients with rheumatoid arthritis. *Clinical & Experimental Immunology*.

[6] Miyara M., Ito Y., Sakaguchi S. (2014). Treg-cell therapies for autoimmune rheumatic diseases. *Nature Reviews Rheumatology*.

[7] Bin Dhuban K., Piccirillo C. A. (2015). The immunological and genetic basis of immune dysregulation, polyendocrinopathy, enteropathy, X-linked syndrome. *Current Opinion in Allergy and Clinical Immunology*.

[8] Marazuela M., García-López M. A., Figueroa-Vega N. (2008). Regulatory T cells in human autoimmune thyroid disease. *The Journal of Clinical Endocrinology & Metabolism*.

[9] Danikowski K. M., Jayaraman S., Prabhakar B. S. (2017). Regulatory T cells in multiple sclerosis and myasthenia gravis. *Journal of Neuroinflammation*.

[10] Battaglia M., Gregori S., Bacchetta R., Roncarolo M. G. (2006). Tr1 cells: from discovery to their clinical application. *Seminars in Immunology*.

[11] Collison L. W., Chaturvedi V., Henderson A. L. (2010). IL-35-mediated induction of a potent regulatory T cell population. *Nature Immunology*.

[12] Han Y., Guo Q., Zhang M., Chen Z., Cao X. (2009). CD69^+^CD4^+^CD25^−^ T cells, a new subset of regulatory T cells, suppress T cell proliferation through membrane-bound TGF-*β*1. *The Journal of Immunology*.

[13] Vitales-Noyola M., Doníz-Padilla L., Álvarez-Quiroga C., Monsiváis-Urenda A., Portillo-Salazar H., González-Amaro R. (2015). Quantitative and functional analysis of CD69+ NKG2D+ T regulatory cells in healthy subjects. *Human Immunology*.

[14] Muñoz-Rodríguez A., Vitales-Noyola M., Ramos-Levi A., Serrano-Somavilla A., González-Amaro R., Marazuela M. (2016). Levels of regulatory T cells CD69^+^NKG2D^+^IL-10^+^ are increased in patients with autoimmune thyroid disorders. *Endocrine*.

[15] Vitales-Noyola M., Oceguera-Maldonado B., Niño-Moreno P. (2017). Patients with systemic lupus erythematosus show increased levels and defective function of CD69^+^ T regulatory cells. *Mediators of Inflammation*.

[16] Zhu J., Feng A., Sun J. (2011). Increased CD4+CD69+CD25- T cells in patients with hepatocellular carcinoma are associated with tumor progression. *Journal of Gastroenterology and Hepatology*.

[17] Dai Z., Turtle C. J., Booth G. C. (2009). Normally occurring NKG2D+ CD4+ T cells are immunosuppressive and inversely correlated with disease activity in juvenile-onset lupus. *The Journal of Experimental Medicine*.

[18] Wang T., Li S., Yang Y. (2016). T helper 17 and T helper 1 cells are increased but regulatory T cells are decreased in subchondral bone marrow microenvironment of patients with rheumatoid arthritis. *American Journal of Translational Research*.

[19] Brand S. (2009). Crohn’s disease: Th1, Th17 or both? The change of a paradigm: new immunological and genetic insights implicate Th17 cells in the pathogenesis of Crohn’s disease. *Gut*.

[20] Gaffen S. L., Jain R., Garg A. V., Cua D. J. (2014). The IL-23-IL-17 immune axis: from mechanisms to therapeutic testing. *Nature Reviews Immunology*.

[21] Kleinewietfeld M., Manzel A., Titze J. (2013). Sodium chloride drives autoimmune disease by the induction of pathogenic TH17 cells. *Nature*.

[22] Zostawa J., Adamczyk J., Sowa P., Adamczyk-Sowa M. (2017). The influence of sodium on pathophysiology of multiple sclerosis. *Neurological Sciences*.

[23] Yang X., Yao G., Chen W., Tang X., Feng X., Sun L. (2015). Exacerbation of lupus nephritis by high sodium chloride related to activation of SGK1 pathway. *International Immunopharmacology*.

[24] Scrivo R., Massaro L., Barbati C. (2017). The role of dietary sodium intake on the modulation of T helper 17 cells and regulatory T cells in patients with rheumatoid arthritis and systemic lupus erythematosus. *PLoS One*.

[25] van der Meer J. W., Netea M. G. (2013). A salty taste to autoimmunity. *The New England Journal of Medicine*.

[26] Hernandez A. L., Kitz A., Wu C. (2015). Sodium chloride inhibits the suppressive function of FOXP3^+^ regulatory T cells. *The Journal of Clinical Investigation*.

[27] Hucke S., Eschborn M., Liebmann M., Herold M., Freise N., Engbers A. (2016). Sodium chloride promotes pro-inflammatory macrophage polarization thereby aggravating CNS autoimmunity. *Journal of Autoimmunity*.

[28] Tanaka T., Okamura T., Miura K. (2002). A simple method to estimate populational 24-h urinary sodium and potassium excretion using a casual urine specimen. *Journal of Human Hypertension*.

[29] Brown I. J., Tzoulaki I., Candeias V., Elliott P. (2009). Salt intakes around the world: implications for public health. *International Journal of Epidemiology*.

[30] Charlton K. E., Steyn K., Levitt N. S., Jonathan D., Zulu J. V., Nel J. H. (2008). Development and validation of a short questionnaire to assess sodium intake. *Public Health Nutrition*.

[31] Canavan J. B., Afzali B., Scotta C. (2012). A rapid diagnostic test for human regulatory T-cell function to enable regulatory T-cell therapy. *Blood*.

[32] Kuek A., Hazleman B. L., Ostör A. J. (2007). Immune-mediated inflammatory diseases (IMIDs) and biologic therapy: a medical revolution. *Postgraduate Medical Journal*.

[33] Sundström B., Johansson I., Rantapää-Dahlqvist S. (2015). Interaction between dietary sodium and smoking increases the risk for rheumatoid arthritis: results from a nested case-control study. *Rheumatology*.

[34] Yi B., Titze J., Rykova M. (2015). Effects of dietary salt levels on monocytic cells and immune responses in healthy human subjects: a longitudinal study. *Translational Research*.

[35] Wen W., Wan Z., Zhou D., Zhou J., Yuan Z. (2017). The amelioration of insulin resistance in salt loading subjects by potassium supplementation is associated with a reduction in plasma IL-17A levels. *Experimental and Clinical Endocrinology & Diabetes*.

[36] Jiang X., Sundström B., Alfredsson L., Klareskog L., Rantapää-Dahlqvist S., Bengtsson C. (2016). High sodium chloride consumption enhances the effects of smoking but does not interact with SGK1 polymorphisms in the development of ACPA-positive status in patients with RA. *Annals of the Rheumatic Diseases*.

[37] Mathis K. W., Venegas-Pont M., Masterson C. W., Wasson K. L., Ryan M. J. (2011). Blood pressure in a hypertensive mouse model of SLE is not salt-sensitive. *American Journal of Physiology Regulatory, Integrative and Comparative Physiology*.

[38] O'Shea J. J., Jones R. G. (2013). Autoimmunity: rubbing salt in the wound. *Nature*.

[39] Croxford A. L., Waisman A., Becher B. (2013). Does dietary salt induce autoimmunity?. *Cell Research*.

[40] Mente A., O'Donnell M., Rangarajan S. (2016). Associations of urinary sodium excretion with cardiovascular events in individuals with and without hypertension: a pooled analysis of data from four studies. *The Lancet*.

[41] Cogswell M. E., Mugavero K., Bowman B. A., Frieden T. R. (2016). Dietary sodium and cardiovascular disease risk–measurement matters. *The New England Journal of Medicine*.

[42] Farez M. F., Fiol M. P., Gaitán M. I., Quintana F. J., Correale J. (2015). Sodium intake is associated with increased disease activity in multiple sclerosis. *Journal of Neurology, Neurosurgery, and Psychiatry*.

